# Challenges to Building a Gene Variant Commons to Assess Hereditary Cancer Risk: Results of a Modified Policy Delphi Panel Deliberation

**DOI:** 10.3390/jpm11070646

**Published:** 2021-07-08

**Authors:** Mary A. Majumder, Matthew L. Blank, Janis Geary, Juli M. Bollinger, Christi J. Guerrini, Jill Oliver Robinson, Isabel Canfield, Robert Cook-Deegan, Amy L. McGuire

**Affiliations:** 1 Center for Medical Ethics and Health Policy, Baylor College of Medicine, One Baylor Plaza, Houston, TX 77005, USA; matthew.blank@bcm.edu (M.L.B.); jmurph46@jhu.edu (J.M.B.); guerrini@bcm.edu (C.J.G.); Jill.Robinson@bcm.edu (J.O.R.); amcguire@bcm.edu (A.L.M.); 2 School for the Future of Innovation in Society and Consortium for Science, Policy & Outcomes, Arizona State University Barrett & O’Connor Washington Center, 1800 I (Eye) Street, NW, Washington, DC 20006, USA; jdgeary@asu.edu (J.G.); bcd@asu.edu (R.C.-D.); 3 Johns Hopkins Berman Institute of Bioethics, Deering Hall, 1809 Ashland Ave, Baltimore, MD 21205, USA; 4 Department of Philosophy, University of Notre Dame, Malloy Hall, Notre Dame, IN 46556, USA; icanfiel@nd.edu

**Keywords:** hereditary cancer, human genetics, data sharing, data resources, privacy and security, equity

## Abstract

Understanding the clinical significance of variants associated with hereditary cancer risk requires access to a pooled data resource or network of resources—a “cancer gene variant commons”—incorporating representative, well-characterized genetic data, metadata, and, for some purposes, pathways to case-level data. Several initiatives have invested significant resources into collecting and sharing cancer gene variant data, but further progress hinges on identifying and addressing unresolved policy issues. This commentary provides insights from a modified policy Delphi process involving experts from a range of stakeholder groups involved in the data-sharing ecosystem. In particular, we describe policy issues and options generated by Delphi participants in five domains critical to the development of an effective cancer gene variant commons: incentives, financial sustainability, privacy and security, equity, and data quality. Our intention is to stimulate wider discussion and lay a foundation for further work evaluating policy options more in-depth and mapping them to those who have the power to bring about change. Addressing issues in these five domains will contribute to a cancer gene variant commons that supports better care for at-risk and affected patients, empowers patient communities, and advances research on hereditary cancers.

## 1. Introduction

An accurate understanding of variants associated with hereditary cancer risk requires access to substantial, representative, well-characterized genetic data [1]. If data are to support scientific and clinical inference, they must be linked to information about the methods used for interpretation and other forms of metadata. The reliability of scientific findings and clinical assessments will sometimes require the ability to track back to the origin of data and information about specific cases. Development of a pooled data resource or network of resources meeting these requirements—a “cancer gene variant commons”—has been hindered by policy issues as well as technical and logistical challenges.

Efforts to develop a data resource to help interpret the significance of variants in genes associated with hereditary cancer risk date back to the launch of the Breast Cancer Information Core (BIC) in 1995. The BIC focused on documenting variations in the *BRCA1* and *BRCA2* genes [2], while the Breast Cancer Association Consortium (BCAC), formed in 2005, compiled data on gene-disease associations [3]. In 2014, the Global Alliance for Genomics and Health (GA4GH) developed the BRCA Exchange to compile variants and corresponding evidence to support clinical risk classifications [4]. As of May 2021, the BRCA Exchange reported over 66,162 variants of the two BRCA genes. New variants of unknown significance continue to be identified for these well-characterized genes, and a large majority (58,717) of those variants remain “not yet reviewed.” Even less progress has been made to compile the data needed to support classification for other cancer genes. Significant work is needed on classification and data sharing to catch up with the progress that has been made in data production.

Further progress will depend on identifying the most important policy issues, identifying and assessing relevant policy options, and bringing this work to the attention of those who have the power to bring about change. Addressing these issues will facilitate the creation of a cancer gene variant commons that leads to better care for those at genetic risk of cancer and for patients whose treatment is influenced by their genotype. A more robust cancer gene variant commons can empower patient communities, advance research on hereditary cancers, inform management of cancer risk, and improve cancer care.

## 2. Modified Policy Delphi Process

The Sulston Project is a National Cancer Institute (NCI)-funded research project (R01 CA237118) examining how to facilitate data sharing and create an effective cancer gene variant commons. The Sulston Project is informed by a panel of 24 experts representing five groups: data contributors and end-users (patients, families, and advocacy organizations); data generators (testing laboratories, both clinical and research); data resources (databases and repositories); data facilitators (data curators/annotators and variant interpreters); and professional data users (genetic counselors, other clinicians, and researchers). That expert panel is participating in a modified policy Delphi process, an iterative, systematic process for converging on findings. The original Delphi method was pioneered by the RAND corporation as a forecasting tool [5,6]; it was later modified to provide expert input to those making policy decisions [7,8]. This paper summarizes the first three rounds of a Delphi process focused on generating policy options for a cancer gene variant commons. This process involved identifying issues that will influence how effectively the cancer gene variant commons operates, ranking those issues according to their importance and the feasibility of addressing them, and then exploring potential policy options to consider. A final phase will focus on evaluating policy options in greater depth, mapping options to entities with the power to act on them, and disseminating findings to those target audiences.

In the first round of the Delphi, we conducted semi-structured interviews with panelists that yielded a preliminary list of 16 statements intended to convey policy issues related to developing a cancer gene variant commons (full list included in the Supplementary Materials). In a survey administered in the second Delphi round, panelists rated each of these issues for their validity, importance, and feasibility, and at the end, identified and ranked the three most important issues from the list. Results of the quantitative analysis identified high-priority issues in five domains: incentives, financial sustainability, privacy and security, equity, and data quality (Table 1). In round three, we convened a virtual full-day meeting of the Delphi panel to participate in guided, structured deliberation focused on generating initial policy options for each high-priority issue.

## 3. Policy Options

Panelists were divided into discussion groups based on panelists’ preferred issues and expertise to consider policy options for each issue (with the exception of privacy and data security, two issues that were combined for discussion). However, we discovered that some policy options were put forward in multiple sessions, which speaks to the interconnectedness of these issues. This was especially true for incentives and financial sustainability; hence, the policy options generated for those two issues are reviewed together.

### 3.1. Incentives and Financial Sustainability

The co-occurrence of policy options for the incentives and financial sustainability issues makes sense because the majority of policy options addressing incentives relate to potential structures for a cancer gene variant commons and the data resources that comprise it that align with different funding models. Many panelists believed that some form of public-private partnership would be necessary for long-term success. One panelist stated “Realistically, [a cancer gene variant] commons cannot rely solely on public support over the long term…” and it will be important to bring “private and public interests together.” (Of note, a public-private partnership model was also discussed as a desirable component of the response to the data quality issue.) The public side, in the form of federal funding, was seen as particularly important initially. However, the National Institutes of Health (NIH) usually funds research through time-limited grants, typically in the range of 4–5 years. Absent changes to this approach, it was thought that the commons could transition to industry and philanthropic funding over time to ensure longer-term financial stability.

Some panelists envisioned a public-private partnership taking the form of a two-tier consortium, with a public tier of data freely available to all qualified users and a premium tier conferring special access or other privileges on contributors of data and/or financial support. This structure would counter commercial disincentives to participation. Panelists discussed a variety of approaches to the premium tier. For example, contributors might be given exclusive access to their own data or to all pooled data for a limited period of time, or an enriched tier of pooled data might be permanently restricted to contributors. Contributors could also be given a voice in governance. Several panelists mentioned the Structural Genomics Consortium (SGC), a not-for-profit organization funded by pharmaceutical companies, governments, and charities, as a successful partial model. The SGC leadership keeps a confidential list of priority molecular targets to study, but the scientific outputs—data on the molecular structures studied and chemical probes—are open access [9,10]. In exchange for their financial support, funders gain influence over the SGC’s research agenda and the right to nominate a member of its Board of Directors. Using a related concept, some described a pay-to-play system where those who “pay” by contributing to the commons financially and/or by depositing data thereby secure the opportunity to “play” in the sense of having a say in the operation of the commons and, potentially, access to an enriched tier of data. One participant noted, “My dream is that you solve the free-rider problem by having a trusted intermediary that sells tickets. If you don’t share, you don’t get the data. You may get a public interpretation of results, but you don’t get to make the decisions about research priorities.” This led to a discussion of a three-tier data access structure analogous to the approaches adopted by the ELIXIR-Beacon network and the Montreal Neurological Institute. One layer of data is freely available, a second layer is available to commons members (certified or registered access), and a third layer contains data that is individually identifiable or otherwise sensitive and is available only as approved by a data access committee (controlled access) [11].

With regard to incentives, another policy option would be to leverage the power of payers and providers (e.g., insurance companies, ordering institutions, genetic counselors) to encourage and facilitate testing by laboratories that commit to contributing results to the commons. An analogous option would be to leverage the power of funders to create professional incentives to share (i.e., you contribute data, you get more funding). As one panelist stated, “There is some ability to select where tests can be run for sequencing. Payers and providers can be the force that makes a difference in the commercial laboratory sector, and in the academic sector it is more the funders.” In the context of a cancer gene variant commons, several commentators have suggested that insurers make inclusion in a preferred laboratory network contingent on laboratory data sharing [12,13]. For scientific contributors funded by grants or contracts, this might entail a process to monitor compliance with the data-sharing plans in their grant applications or contracts, and applicable replication standards [14]. The NIH Policy for Data Management and Sharing disappointed some commentators by not requiring sharing and failing to integrate consideration of data-sharing plans into the review process [15]. The final policy, which will take effect in 2023, does state that non-compliance with an approved plan may be taken into account in future funding decisions [16]. Some funders in the cancer space are more exacting; for example, Susan G. Komen requires funded researchers to share data [17], the Parker Institute for Cancer Immunotherapy and the Chan Zuckerberg Initiative have developed extensive data-sharing agreements, and the NCI Cancer Moonshot program gives funding priority to researchers with plans that ensure maximal sharing [18].

Some panelists believed it was important to consider how to augment existing resources rather than adopt a goal of building new resources from scratch. This involves thinking in terms of a federated model, with linkages among many data resources, which allows for considerable pluralism but seeks to ensure some standardization and interoperability among resources and incorporates design features intended to align interests and incentives among public/non-commercial and commercial partners. Another option discussed, consistent with pluralism, was viewing data resources as having a delineated lifespan, rather than aspiring to a commons as a unitary permanent repository. One panelist stated, “Our objective should not be to create a single, global data commons that lives on forever, but a sustainable structure that allows for individual data commons [resources] to form around particular tools and technologies and share that data—some of which will be cycled out over time.”

### 3.2. Privacy and Security

There are two overarching approaches (not mutually exclusive) to addressing the intertwined challenges of privacy and security through legislation. One approach would focus on legislative solutions that strengthen data protection, that is privacy legislation that would address the limitations of the Common Rule and the Health Insurance Portability and Accountability Act (HIPAA) Privacy Rule. One panelist mentioned the European Union’s General Data Protection Regulation as a possible starting point for major legislative reform. Panelists as a group supported meaningful sanctions for violations and considered enforcement mechanisms such as a private right of action that would enable individuals to sue for damages. Another approach would protect participants in the commons from any harm associated with breaches. One panelist succinctly stated the rationale: “If you contribute data (altruistically) then it shouldn’t be…weaponized against you.” In particular, more comprehensive anti-discrimination legislation would address the limitations of the Affordable Care Act and the Genetic Information Nondiscrimination Act. Specifically, the absence of protection from discrimination in applying for long-term care insurance, disability insurance, and life insurance was noted. While state laws have the potential to address gaps in federal law, the current patchwork does not achieve the benefits of a consistent, reliable floor of protection across the US [19,20].

In stressing the need for new legislation, panelists cited the inadequacies of relying on consent or individual contracts (such as data use agreements) to address privacy and security challenges. One panelist commented: “Individual contracts prevent institutions from doing something terrible or if they do something terrible, they get penalized. But the problem is if something happens and someone does something wrong, the institution gets penalized, but the person’s data may already be compromised and there is still no protection for the patient. The overall regulatory environment [i.e., the legislative landscape] has to change.” Responding to developments including the 2017 revisions to the Common Rule that allow for broad consent to unspecified future use of de-identified data [21], another panelist stated that “too much is being put on broad consent.” The common thread is a belief that individuals agreeing to have their data shared and used for research purposes cannot fully appreciate all risks, including some risks of harm that are currently unforeseeable, nor should they be expected to assume those risks. Hence, strengthened anti-discrimination legislation is necessary to safeguard individuals (and communities) contributing to the commons against an important class of potential harms.

The panelists also discussed addressing privacy concerns through a data ethics board. Such a board (or boards, to accommodate a federated data structure) could serve as a gatekeeper to data, for example, establishing criteria for data access tiers, and monitor data uses. Sage Bionetworks, for example, has formal access policies, and those using its data resources know their uses are monitored [22]. A data ethics board would also play a role in establishing procedures for enforcement of rules surrounding data and imposition of sanctions in the case of violations. In addition, panelists described putting funds aside to provide compensation to individuals affected by rule violations committed by researchers and other legitimate users or security breaches engineered by third-party attackers. One possible implementation strategy in the former case would be bonding researchers who use data from the commons, especially researchers outside of traditional institutional accountability structures. Surety bonds are a form of insurance that compensates third parties (typically, customers, suppliers, or taxpayers) in the event of some person or entity’s failure to fulfill specified obligations.

### 3.3. Equity

A major goal of a cancer gene variant commons is the inclusion of underrepresented communities and advancing equity. However, this can be a challenge given histories of exploitation by biomedical researchers and continuing inequities in health care. Panelists generated a series of policy options to address systemic and structural biases that undermine efforts to create an equitable and representative commons.

A direct contributor to exacerbating existing health inequities is the deficit of genetic data from individuals with non-European ancestry in data resources (96% European descent versus 4% non-European descent) [23,24]. The significance of founder mutations is illustrated in a comprehensive study of breast and ovarian cancer-related *BRCA1/2* mutations specific to different geographic and ethnic regions in Europe. Pathogenic founder mutations that drive late-onset disease conditions are not subjected to negative selective pressure and emerge from population bottlenecks with founder mutations fixed within a population’s genome [25]. Because of the vast amount of European genetic data, researchers could accurately identify point, frameshift, duplication, and insertion/deletion *BRCA1/2* founder mutations—a feat only accomplished through expansive sampling [26]. While some *BRCA1/2* founder mutations have been identified in Asian populations [27,28], the most extensive research centers on populations of European ancestry. This means that rarer pathogenic variants are unidentified in non-European populations, increasing the return of variants of uncertain significance and risks of misdiagnosis in individuals from populations that have been less tested [23,24,28]. Moreover, identifying an individual’s accurate (and comprehensive) polygenic background dramatically increases the accuracy of calculations of the potential risk of inheriting a monogenic variant, for example in *BRCA1/2* [29], which amounts to an immediate and additional disadvantage to populations underrepresented in biobanks and databases [29]. While the gathering of data from European ancestry populations may not be due to deliberate exclusion of non-European populations, intentional strategies and policies will need to be employed for inclusion, especially considering the well-earned distrust of many underrepresented groups and access barriers limiting clinical testing of these groups [30].

A policy option that gained significant traction among the panelists was to conduct thorough community needs assessments to identify community priorities and create greater alignment between funding and those priorities. A poignant moment in the discussion involved a description of a previous research study when “[researchers] went to Guatemala and said to the community, ‘We have money to research Topic X. What do you think about Topic X?’ and the community said, ‘Well, we would like you to use that money instead to buy soap for our public hospital.’” This panelist continued by noting the “disconnect between what white academics are interested in researching (and what the NIH is interested in funding), and what communities self-identify as their priority.” Importantly, the example suggests that funders and investigators should adopt a vision in which meeting high-priority community needs is a necessary component of advancing research goals. There is guidance that already exists on how to do this effectively, some specific to indigenous groups with sovereign governments [31,32,33,34], and some more broadly relevant to communities without formal governance structures [35].

The sense of the Delphi panel was that engaging communities in bi-directional dialogue focused on transparency and trustworthiness must precede other options for addressing equity. However, several other options were discussed, including more creative and inclusive approaches to data governance (e.g., multidisciplinary oversight that includes advocates, lawyers, technical specialists, and political scientists) and adoption of measures that reduce risks of harm or compensate individuals who experience harms (as was discussed above under “Privacy and Security”), since many members of underrepresented communities are particularly vulnerable to harm. The panelists also felt that it would be essential to adopt a multidisciplinary approach to engagement, which would include “anthropologists and other social scientists [to] help frame these conversations [with communities and] build them into projects.”

In addition, the panel discussed providing compensation/giving back to participant communities as an acknowledgment of their contribution and to incentivize participation. Finally, the panelists noted a role for journals in addressing inequities. One stated that “journals should require complete transparency from their authors by requiring statements of effort to include diverse data, why the authors chose a particular dataset or recruited a specific population (and excluded others). Authors should also attest to the pedigree of the dataset to help identify unethically obtained [data].”

### 3.4. Data Quality

As a foundation for discussion of data quality, panelists stressed the importance of clearly defining the contents of the commons, which would streamline the process of developing standards for maintaining quality data. While the purpose of the Delphi was not to identify technical solutions, the Delphi panel noted that establishing technical standards would be critical. Once the content of the commons is clear, one option for increasing data quality would consist of tying positive incentives associated with data contribution (e.g., preferred status with payers and providers, funding) to data quality, including compliance with standards.

Beyond technical standards for the data itself (i.e., FASTA reads, assembled sequences, variant calls, etc.), the issue of data quality can be addressed by facilitating interoperability among databases and creating a multi-user-friendly API-based platform. This includes a platform for layers of stakeholders to easily deposit, search, and interpret data, as the “easier [it is] for people to do things (conform to standards for data quality, share data), [the] more likely [they are] to do them.” Additionally, standardization of data requires some degree of central governance to enforce compliance with standards. Panelists expressed the need for a trustworthy governance structure to maintain the integrity of the data, whether in the form of a central gatekeeper, possibly with a federal government affiliation, or professional society oversight. For example, a panelist stated, “Who builds it and who maintains it, in the long run, is critical. This requires a centralized, federally-supported structure.” This is a clear example of connection across issues, as it leads back to the discussion of financial sustainability.

## 4. Conclusions

Our Delphi panelists identified high-priority policy issues that are critical to the construction of effective cancer gene variant commons in the domains of incentives, financial sustainability, privacy and security, equity, and data quality. We have also laid out an initial set of policy options for addressing these issues, noting relationships across domains and issues. With this commentary, we hope to stimulate wider discussion of these issues and lay a foundation for further work evaluating policy options in greater depth and mapping them to actors with the power to bring about needed change. The issues described are complex and challenging—if they were simple and easy, they would already have been resolved. Yet we have emerged from these deliberations with experts optimistic about the eventual creation of a cancer gene variant commons that empowers patient communities and advances research on hereditary cancers, leading to significant improvements in human health and well-being.

## Figures and Tables

**Table 1:** Domains and Related High-Priority Issue Statements *.

Incentives	Some entities that generate data are not sharing data because of countervailing incentives and values. (For example, some might not share due to professional incentives or requirements that are not aligned with sharing, such as academic promotion standards, or because they believe that not sharing gives them a competitive advantage.)
Financial Sustainability	The commons has characteristics of a public good, which makes ensuring long-term sustainability challenging because of the lack of market incentives.
Privacy and Security	Trust in the security of a commons is difficult to build given that privacy breaches can never be completely eliminated and laws/regulations/norms protecting privacy change over time. A wealth of linked data is necessary to solve complex problems (for example, phenotypic and associated data), but then the data become more identifiable, and privacy risks increase (especially, for example, for smaller populations like Tribal groups and patients with rare diseases).
Equity	The commons should not perpetuate inequities in health care or create new ones. Uses should also aim to address inequities. (For example, using commons data to develop a diagnostic test that is most suitable for individuals of European ancestry would likely exacerbate existing health disparities.)
Data Quality	Shared data are of variable quality, and there is no consensus regarding how to monitor and assess the quality of data sources.

* Final issue statements reflect alterations made by panelists during group deliberation.

## Data Availability

De-identified data presented in this study are available on request from the corresponding author. The data are not publicly available due to confidentiality considerations.

## Supplementary Materials

The following are available online at https://www.mdpi.com/article/10.3390/jpm11070646/s1, Table S1: List of 16 original issue statements.
