# Supplementary material for: Challenges to Building a Gene Variant Commons to Assess Hereditary Cancer Risk: Results of a Modified Policy Delphi Panel Deliberation
Source: J Pers Med. 2021 Jul 8;11(7):646. doi: 10.3390/jpm11070646 (PMC8305920; doi:10.3390/jpm11070646)
Supplement: Supplementary file 1 [file jpm-11-00646-s001.zip › jpm-1215759-supplementary.pdf]

Issue Statements	
1	Some entities that generate data are not sharing it because of countervailing incentives and values. (For example, some might not share due to professional incentives or requirements that are not aligned with sharing, such as academic promotion standards, or because they believe that not sharing gives them a competitive advantage.)
2	There is disagreement and confusion regarding who “owns” data resources that make up the commons and what rights or interests are associated with ownership, which may frustrate efforts to achieve consensus about governance of those resources.
3	Data resources in the commons are collected and shared across multiple jurisdictions that have different laws/regulations/norms related to privacy, consent, and governance.
4	There is disagreement about whether data in the commons should be available only to traditional researchers and clinicians or should also be accessible in some form to patients and others who are not affiliated with scientific institutions (for example, “citizen scientists”).
5	Substantial effort will be required to explain the purpose and use of a genomic commons to the public and participants, including potential implications for patients.
6	The commons should not perpetuate inequities in health care, or create new ones. Uses should also aim to address inequities. (For example, using commons data to develop a diagnostic test that is most suitable for individuals of European ancestry would likely exacerbate existing health disparities.)
7	There is disagreement regarding the nature and extent of the problem of lack of diversity or representativeness of data, and who is responsible for addressing it, which limits progress in this area.
8	Substantial effort will be required to earn the trust of individuals and communities who should be able to participate in the commons but are not inclined to do so as a result of their past exploitation by researchers.
9	Trust in the security of a commons is difficult to build given that privacy breaches can never be completely eliminated and laws/regulations/norms protecting privacy change over time.
10	A wealth of linked data is necessary to solve complex problems (for example, phenotypic and associated data), but then the data become more identifiable and privacy risks increase (especially, for example, for smaller populations like Tribal groups and patients with rare diseases).
11	It is important to engage representatives of multiple stakeholder groups in developing and implementing the rules for the commons, but their competing interests can lead to gridlock.
12	There is a lack of trust between stakeholder groups owing at least in part to divergent objectives and motivations (for example, the BRCActivist community and researchers).
13	It is difficult to ensure compliance with rules that are adopted to govern the commons.
14	The commons has characteristics of a public good, which makes ensuring long-term sustainability challenging because of lack of market incentives.
15	There are benefits associated with the involvement of commercial interests in the commons (with the potential to profit downstream), but that involvement has the potential to decrease public trust in and therefore support of the commons
16	Shared data are of variable quality and there is no consensus regarding how to monitor and assess the quality of data sources.
